# Serotonin and Noradrenaline Reuptake Inhibitors Improve Micturition Control in Mice

**DOI:** 10.1371/journal.pone.0121883

**Published:** 2015-03-26

**Authors:** Marco Redaelli, María Jimena Ricatti, Marialaura Simonetto, Mirko Claus, Maurizio Ballabio, Antonio Caretta, Carla Mucignat-Caretta

**Affiliations:** 1 Department of Molecular Medicine, University of Padova, Padova, Italy; 2 National Institute of Biostructures and Biosystems, Roma, Italy; 3 Cell Biology and Neuroscience Institute, University of Buenos Aires—National Scientific and Technical Council (UBA-CONICET), Buenos Aires, Argentina; 4 Ronzoni-Villa Foundation, Seregno, Italy; 5 Pharmaceutical Department, University of Parma, Parma, Italy; University of Pittsburgh School of Medicine, UNITED STATES

## Abstract

Poor micturition control may cause profound distress, because proper voiding is mandatory for an active social life. Micturition results from the subtle interplay of central and peripheral components. It involves the coordination of autonomic and neuromuscular activity at the brainstem level, under the executive control of the prefrontal cortex. We tested the hypothesis that administration of molecules acting as reuptake inhibitors of serotonin, noradrenaline or both may exert a strong effect on the control of urine release, in a mouse model of overactive bladder. Mice were injected with cyclophosphamide (40 mg/kg), to increase micturition acts. Mice were then given one of four molecules: the serotonin reuptake inhibitor imipramine, its metabolite desipramine that acts on noradrenaline reuptake, the serotonin and noradrenaline reuptake inhibitor duloxetine or its active metabolite 4-hydroxy-duloxetine. Cyclophosphamide increased urine release without inducing overt toxicity or inflammation, except for increase in urothelium thickness. All the antidepressants were able to decrease the cyclophosphamide effects, as apparent from longer latency to the first micturition act, decreased number of urine spots and volume of released urine. These results suggest that serotonin and noradrenaline reuptake inhibitors exert a strong and effective modulatory effect on the control of urine release and prompt to additional studies on their central effects on brain areas involved in the social and behavioral control of micturition.

## Introduction

Micturition disorders cause profound distress and may involve central and peripheral mechanisms. An imbalance in noradrenaline and serotonin is involved in both components: reuptake inhibitors of these neurotransmitters may improve micturition control, although urinary hesitancy and retention are side effects of antidepressant administration [1, 2].

The storage and release of urine are complex functions necessary for both survival and socially appropriate interactions. They require a complex coordination of incoming sensory inputs and descending cortical modulation in pontine centers. Coordination is necessary to finely tune the autonomic activity directed to bladder and urethra, via sympathetic and parasympathetic nerves, and the lumbosacral motoneurons directed to striated sphincters and muscles of the pelvic floor [3]. The activity in motoneurons of Onuf’s nucleus is modulated by both serotonin and noradrenaline, which facilitate glutamatergic activation and hence prevent accidental bladder voiding when increasing abdominal pressure [4]. Serotonin facilitates the sympathetic storage reflex, while inhibiting the parasympathetic voiding activity.

Imipramine, a serotonin reuptake inhibitor, was the first tricyclic drug used for the treatment of incontinence. Because of its sympathomimetic and anticholinergic properties, it may decrease the bladder contractility, by acting also as antagonist on muscarinic receptors [5,6]. Moreover, it increases striated urethral sphincter contractions [7]. Imipramine is readily metabolized to its main active metabolite, desipramine, which acts as a selective noradrenaline reuptake inhibitor. Therefore, clinical effects of imipramine involve two main actions, on serotonin and noradrenaline reuptake.

Duloxetine is a reuptake inhibitor for both serotonin and noradrenaline [8, 9]. It was the first drug licensed for stress incontinence in 2004 in the European Union, due to its ability to increase bladder capacity and activity in sphincters. Noteworthy, it has no effect on bladder contractile activity during micturition, because its action is only possible in combination with glutamatergic excitation [10]. Its patent protection terminated recently, making it a cost-effective therapeutic option under specific circumstances [11].

The aim of the present work is to test the efficacy of two clinically used tricyclic molecules (imipramine and duloxetine) and their main metabolites (desipramine and 4-hydroxy-duloxetine, respectively) on an animal model of overactive bladder (OAB), induced in an outbred mouse strain. We stimulated bladder overactivity by injecting cyclophosphamide in CD-1 mice, and showed that this can reliably induce an increase in micturition. We then evaluated micturition performance after administering one of four antidepressant molecules: all of them acted by reducing micturition acts, without inducing overt sign of toxicity in the urothelium.

## Materials and Methods

### Pharmacological treatments and behavioral studies

All experiments were carried out in strict accordance with the European law (EU Directive 2010/63/EU) on animal experiments and welfare, and were approved by the competent authorities (local ethical committee and Italian Ministry of Health permit number: 97/2008B-77–08). All efforts were made to minimize discomfort. Mice were kept at 24±1°C, 60% humidity, 12:12 hours light on starting at 6:00 a.m., with mouse food chow (Altromin, Rieper, Bolzano, Italy) and water always available. The same experimenter blind to experimental condition performed all the procedures in the same room. The cyclophosphamide (CYP) OAB model implemented in C57 inbred mice by Boudes and colleagues [12] was validated in outbred CD1 mouse strain by treating seven four-month old male mice (4 i.p. injections 40 mg/kg CYP, one every 48 hours; according to [12]) and five controls with saline (9 g/L NaCl, same time schedule). The CYP dosage was the lowest that increased micturition without altering diuresis [12]. The CD-1 strain was used because its micturition behaviour under various physiological conditions has been already described [13]. The voiding pattern (latency to the first micturition, number of droplets, and urine spot area) was simultaneously measured 24 hours after the last CYP injection at 4:00 p.m. Each mouse was put for 30 minutes in a cage (20x27x18 cm) with the floor covered with a paper sheet lined with plastic film (BenchGuard cat°BG60E) to retain all urine drops. The papers were dried for 48 hours at room temperature, then transilluminated with a UV source to highlight the urine spots and the images were acquired with a digital camera. ImageJ software (version 1.47, NIH, USA) was used to count the number of pixels within the perimeter of each urine spot [14], which was then converted into urine volume (see S1 Fig.).

Subsequently, the effects of four different molecules were tested in the OAB model (see S2 Fig.): imipramine 20 mg/kg [15], desipramine 20 mg/kg [16], duloxetine 2 mg/kg [17] and its metabolite 4-hydroxy-duloxetine 2 mg/kg [18, 19]. For two consecutive days, starting 24 hours after the last CYP injection, OAB induced mice (n = 35 CD1 males, 4 month old, 7 in each group, mean body weight 50.9 ± 0.46 g at the beginning of the treatment, and 51.3 ± 0.37 g at sacrifice) were injected i.p. with saline as control, or with one of the molecules in 100 μl saline. Their voiding pattern was monitored for three days: 24 hours after the last CYP injection but before the antidepressant injection (T0), 24 hours later but before the second treatment (T24), and 24 hours later (T48), before sacrifice. Voiding behaviour was observed as above for 30 minutes, recording the latency to the first micturition, the number of urine spots and the total spot area to calculate the voided volume (Cavaggioni et al., 2008; Supporting Information S1). The last day the animals were sacrificed with excess anesthesia (xilazine 60 mg/kg and ketamine 225 mg/kg body weight i.p.) after monitoring the voiding pattern for 30 minutes and the bladders explanted for histological analysis.

### Histology

After dissection, the bladders were fixed with 4% (w/v) paraformaldehyde in 0.1 M phosphate buffer, pH 7.4 (PBS) for one hour at room temperature and then washed with PBS. All the bladders from the five groups of CYP-treated animals and from an untreated age, sex and weight-matched group were dehydrated in ethanol, cleared in xylene and then embedded in paraffin wax. Bladders were cut in 7 μm sections parallel to the equatorial plane. Alternate bladder sections were stained with routine haematoxylin and eosin to identify the bladder layers to evaluate its overall morphology and to measure the thickness of the urothelium, or with bis-benzimide in PBS (1:1000, Sigma) to visualize nuclei [20].

Images (300 dpi) were obtained using a Leica DMR epifluorescence microscope (Leica Microsystems AG, Wetzlar, Germany) equipped with a Leica DC 100 camera and software, with a 20x objective (Leica HC PL Fluotar NA: 0.50). The digital images were processed with Adobe Photoshop CS6 software. Quantitative analysis was performed with ImageJ software. A microscopic field (20x magnification) was selected from each section (N = 7 to 12 sections per mouse) within the urothelium, to estimate its thickness. The measurement (μm) was performed from the very basal cellular layer to the apical cellular layer of the urothelium, considering the measured distance as a line with perpendicular orientation relative to the basal membrane. For each mouse, at least 15 measurements were selected and averaged, excluding those made on the most curved area of the urothelium. All the images were analysed by the same examiner (MJR), who was blind to the experimental group.

### Statistical analysis

Data (reported in S1 Data, S2 Data, S3 Data, and S5 Data) are presented as mean±SEM unless otherwise stated. The validation data were analyzed with t-test (untreated vs. CYP-treated mice), while the body weight data underwent a bivariate mixed-design ANOVA (Treatment: control vs. CYP-treated; Day of treatment: day 0, 2, 4, 6), followed by Duncan’s *post hoc* test.

The latency to the first micturition, the number of drops and the urine volume estimated from total drop area were analyzed with a bivariate mixed-design ANOVA for the factors Treatment (saline, imipramine, desipramine, duloxetine and 4-OH duloxetine) and Time (T0: before the first antidepressant injection, T24: 24 hours after the first injection, T48: 48 hours after the first injection), and with Duncan’s *post hoc* test to compare treated mice to saline group at the same time point. Since the same time elapsed from the last CYP injection for every group (saline- or antidepressant-treated), this may represent a better control than values from the same-group before injection of the drug.

The histology data were analyzed with a monovariate ANOVA (Treatment: No CYP, CYP followed by: saline, imipramine, desipramine, duloxetine or 4-OH-duoxetine), followed by Duncan’s *post hoc* test. The one-side significance level was set at p<0.05.

## Results

### Validation of the OAB model

**Fig 1:**
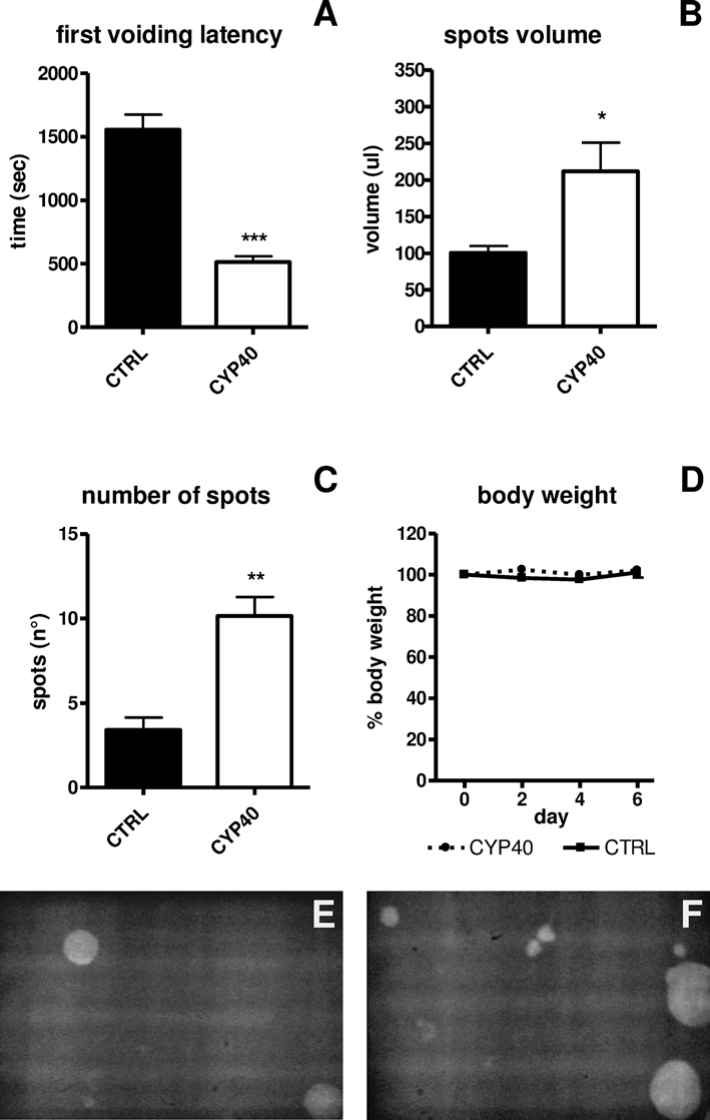
Voiding behaviour of CYP-injected mice (n = 7) in comparison with a saline injected group (n = 5). A, B, C: data describing the micturition pattern: first voiding latency (A), urine volume (B) and number of urine spots (C). D: body weight during the experiment. E, F: Representative UV images of urinary spots of a control mouse (E) in comparison with a CYP-treated mouse (F). Data are presented as mean±SEM. ANOVA and Duncan *post hoc* test, *: p<0.05, **: p<0.005, ***: p<0.001.

The OAB model was tested and validated on CD-1 mice (Fig. 1). A statistically significant reduction in the first voiding latency, t(10) = 9.297, *p*<0.0001, and increase in voiding, both number of spots t(10) = 4.550 *p* = 0.001 and volume of released urine, t(10) = 2.337 *p*<0.05, was apparent in CYP-treated mice, as expected. The mice body weight did not change during the treatment.

### Antidepressants revert the CYP-induced increase in urine release

**Fig 2:**
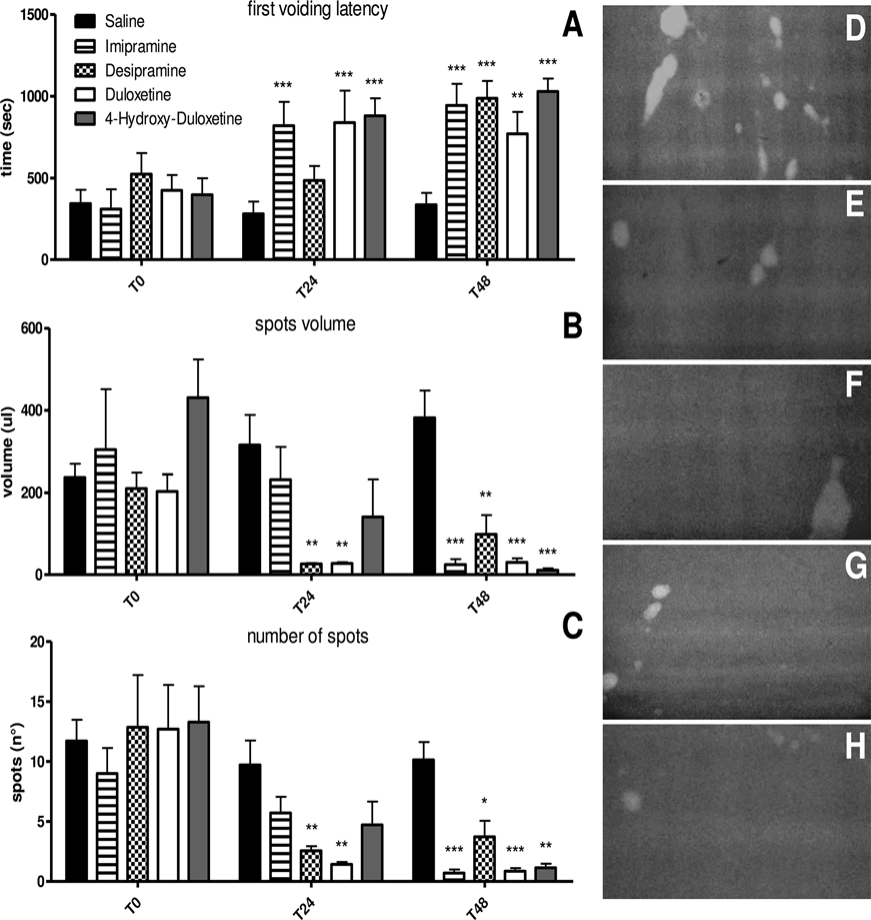
Voiding behaviour of CYP-injected mice treated with saline or antidepressants. A, B, C: data describing the micturition pattern: first voiding latency (A), urine volume (B) and number of urine spots (C). Data are presented as mean+SEM. ANOVA and Duncan *post hoc* test (comparison with saline group) *: p<0.05, **: p<0.005, ***: p<0.001. D, E, F, G and H: representative UV images of urinary spots collected 48 hours after the beginning of the antidepressant treatment: control CYP-injected mouse (D), desipramine (E), imipramine (F) duloxetine (G) and 4-hydroxy-duloxetine-treated mouse (H). The images show a decrease in voiding behaviour in antidepressant-treated mice.

After validation of the CD1 mouse OAB model, the effect of antidepressants was evaluated in 35 OAB-induced mice (Fig. 2). The latency to the first micturition was different according to Time, F (2, 60) = 24.427, *p*<0.0001, with a significant increase in latency at each time point (Duncan’s test, Fig. 2A). In addition, the Treatments were different, F (4,30) = 4.144, *p*<0.01, since only saline-treated mice showed a significantly shorter latency compared to all the other treatments, which did not differ among each other. These differences are best explained by the significant interaction Time x Treatment, F (8,60) = 3.680, *p*<0.002. The *post hoc* test showed that at T0 the latencies did not differ among groups. While saline-treated mice did not change their latency to the first micturition at the three time-points, the administration of imipramine, duloxetine and 4-OH-duloxetine increased latency significantly at T24 and T48, while desipramine increased it only at T48. The volume of voided urine changed across treatments, F (4,30) = 5.388 *p*<0.005, with a significant decrease for imipramine, desipramine, duloxetine and 4-OH-duloxetine compared to saline. It also changed across Time F (2,60) = 9.471, *p*<0.0005, with a significant decrease in volume at T24 and T48 (Duncan’s test, Fig. 2B). The significant Time x Treatment interaction F(8,60) = 3.249, *p*<0.005 showed that at T0 the groups did not differ, while desipramine and duloxetine induced a decrease in voided urine already at T24, and all four antidepressants induced a decrease at T48. The number of urine spots differed according to time, F (2,60) = 38.115, p<0.0001, with more spots at T0 compared to T24 and T48, which did not differ between each other (Duncan’s test, Fig. 2C). The significant interaction Time x Treatment, F (8,60) = 2.583, p<0.02, showed that at T0 all groups produced a high yet similar number of urine spots. Then, the number of spots did not change in saline-treated mice across time, while it decreased at T24 for desipramine and duloxetine and at T48 for all the four antidepressants. Therefore, antidepressant-treated groups are characterized by a relevant reduction in voiding behaviour more evident after 48 hours, by showing an increase in the first void latency and a reduction in the number of droplets and in the urine volume in comparison with the control saline group. The changes in the voiding pattern support a broad efficacy of treatments in OAB.

### Histology

**Fig 3:**
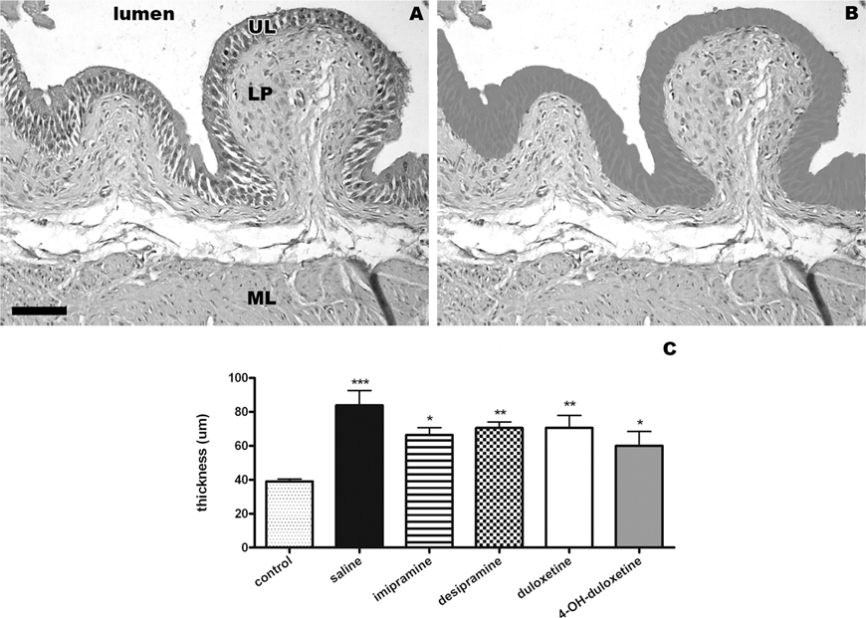
Transverse bladder sections stained with hematoxylin and eosin for mucosa thickness measurement. The bladder mucosa (UL: urothelium layer, LP: lamina propria) and the muscular layer (ML) are shown. Scale bar = 100 μm, 20X magnification. A and B: mucosa of a CYP-treated mouse in contact with the lumen at the top and the entire muscular layer at the bottom. A representative bladder section shows the morphology of the tissue (A) and the precise area in grey (B) corresponding with the urothelium, where its thickness was measured. C: thickness of the urothelium (μm) plotted across experimental groups. Control represents bladders of untreated mice, while all the other bars refer to CYP-treated mice, injected with saline or antidepressant. Data are presented as mean+SEM compared with control group, ANOVA and Duncan *post hoc* test * p<0.05, ** p<0.005, *** p<0.001, n = 7 for each experimental group.

Measurements on bladder sections were carried out to evaluate the cyto-architecture of the mucosa. The staining did not show lymphocytes infiltration in the urothelium or lamina propria. Moreover, no additional cardinal sign of edema or inflammation was found in the mucosa layer. Lastly, no necrotic lesion through the bladder wall was found under our experimental conditions. However, some morphological changes were apparent in the urothelium (Fig. 3): the thickness of urothelium was different F(5,36) = 5.713, p<0.001 among groups, with all CYP-treated groups having a larger urothelium than the untreated controls.

## Discussion

The central nervous system is the site of integration and proper implementation of storage and voiding reflexes, to coordinate the urinary bladder and urethra contractions [21]. Therefore, centrally acting drugs may be necessary for proper management of voiding dysfunction [22]. Moreover, decision about switching from storage to voiding is dependent on social context and is strongly affected by mood [23]. In turn, voiding may affect mood and behavior, in particular in cases of dysfunction [24]. Consequently, micturition is strictly related to higher functions and behavioral control, so that urinary incontinence may benefit from treatments, like antidepressants, directed to improve not only the bladder function, but also the whole autonomic, neuromuscular and integrated behavioral control [25]. Animal studies indicate that spontaneous bladder activity is high in newborns and old rats [26], suggesting that central control may become more relevant to overcome spontaneous activity.

Urine storage involves a spinal reflex that activates somatic motoneurons and sympathetic efferents to the urethra, as well as a central inhibition of voiding. On the contrary, voiding requires a parasympathetic reflex integrated by the pontine micturition center and contextual inhibition of somatic and sympathetic activity [27]. The pontine micturition center coordinates autonomic and somatic activity through the constant inflow of sensory afferents that monitor bladder filling [28], under the modulating influxes of many neurotransmitter systems [29]. In both rats and humans the midbrain periacqueductal gray, which receives bladder sensory afferents, may activate the pontine micturition center to induce voiding [30, 31]. Afferents directed to the periacqueductal gray are subsequently mapped in the insula and monitored by the anterior cingulate gyrus, while the prefrontal cortex is involved in voiding initiation, by relieving its inhibition to the pontine micturition center [32, 33].

Noradrenaline, serotonin and dopamine are the most important modulatory neurotransmitters of this function.

The rationale for duloxetine use in incontinence relies on the modulatory actions of serotonin and noradrenaline on the neural control centers of the bladder and urethra, located in the sacral spinal cord, to promote storage and prevent urine leakage, via the contraction of the external urethral sphincter [34], and on the direct centrifugal inhibition exerted by serotonergic fibers from the raphe nuclei on sensory afferents, resulting in reduced bladder activity [35]. Moreover, poor control of the urinary bladder is associated with insufficient activation of the orbitofrontal cortex, suggesting a central site for drugs aimed at improving micturition function [36]. Acute administration of imipramine and desipramine increases the threshold for the spinal voiding reflex, while chronic administration affects central components [37]. Duloxetine may help to treat overactive bladder in complex neurologic pathologies, either in the presence of depression or not [38–40].

In order to elucidate the complex effect of pharmacologically active molecules, it is necessary to use animal models that closely match the characteristics of the human disease. Micturition disorders were mostly studied in acute animal models, the most common model being the birth trauma, which refers to an acute event [41]. However, in adult humans overactive bladder develops as a consequence of chronic conditions and rarely involves selected nerve lesions. A single chronic OAB mouse model was developed [12]: we refined it to evaluate the effect of antidepressant agents. Our data show that it is possible to induce a model of OAB also in outbred CD-1 mice. These may represent a cost-effective choice, compared to the inbred C57 strain originally used. In addition, the micturition behaviour for CD-1 mice is well known, since this strain is currently used for behavioural studies on urine countermarking [13, 14]. Moreover, the behavioural phenotype of CD-1 mice as a model for depression has been described [42]. The present OAB protocol does not interfere with major determinants of normal behaviour, therefore it allows the sensitive detection of modifications related to the micturition behaviour. Lastly, it allows the recognition of morphological changes across the bladder layers, since it causes no major tissue damage.

Our data show that 40 mg/kg CYP dosage was effective in the induction of OAB condition in CD-1 mouse strain, while a higher dosage is currently employed for mimicking bladder pain syndrome [43]. Body weight, behavioural assessment and histology confirmed that CYP injection was without relevant side effects, as previously reported for C57/Bl6J mice [12]. After 48 hours, each of the four pharmacologically active agents was able to relieve signs of urgency, as demonstrated by increased latency to the first micturition and decreased spots number and volume of released urine. Only minor differences were detected between the molecules, in particular on the time-course of their effects. No difference was present on the magnitude of the effects 48 hours after the beginning of the treatment. These data suggest that the possible scale-down of dosage would permit the appearance of variations in the efficacy of the treatment, as well as prolonged treatments, as it happens for human patients, would allow the emergence of a more complete picture of the peripheral and central effects.

Given the complex central and peripheral effects of antidepressant molecules on micturition, the present results could be complemented by studies aimed at elucidating central action of antidepressant on micturition control, to gain knowledge that may improve their clinical use beyond their antidepressant activity.

## Supporting Information

S1 Data Raw data for Fig. 1. (PDF)

S2 Data Raw data for Fig. 2. (PDF)

S3 Data Raw data for Fig. 3. (PDF)

S4 Data Raw data for S1 Fig. (PDF)

S1 Fig Calibration curve used to calculate the volume of voided urine. Different volumes of male mouse urine (from 1 to 750 microliters) were spotted on the Benchguard paper sheet (the same type used for urine drop analysis) and processed in the same way: they were left for 48 hours at room temperature, then UV transilluminated and photographed. The total number of pixels within urine spots was calculated with ImageJ software, then converted into volume of voided urine according to the equation: y = 445.39x + 2710.3. This gives a linear fit R^2^ = 0.9964, according to the calibration curve.
(PDF)

S2 Fig Experimental layout. Mice were injected with CYP (40 mg/kg), one injection every 48 hours, for four times. 24 hours after the last CYP injection mice were tested for micturition (T0) and then were given a control (saline) or antidepressant injection (see text for dosages). 24 hours later, they were tested again (T24) and then were given the second injection. After additional 24 hours, they were tested for micturition behavior for the third time (T48).
(PDF)

## References

[1] Verhamme KM, Sturkenboom MC, Stricker BH, Bosch R. Drug-induced urinary retention: incidence, management and prevention. Drug Safety. 2008;31: 373–388. 18422378 10.2165/00002018-200831050-00002

[2] Wade AG, Crawford GM. Urinary flow and urinary symptoms in elderly males exposed to either escitalopram or duloxetine. Curr Med Res Opin. 2010;26: 1031–1035. 10.1185/03007991003661877 20199139

[3] Thor KB, de Groat WC. Neural control of the female urethral and anal rhabdosphincters and pelvic floor muscles. Am J Physiol Reg Integr Comp Physiol. 2010;299: R416–R438. 10.1152/ajpregu.00111.2010 20484700 PMC2928615

[4] Thor KB. Targeting serotonin and norepinephrine receptors in stress urinary incontinence. Int J Gynecol Obstet. 2004;86: S38–S52. 10.1016/j.ijgo.2004.04.028 15302566

[5] Wein AJ. Pharmacologic options for the overactive bladder. Urology. 1998;51 2A Suppl: 43–47. 9495736 10.1016/s0090-4295(98)90009-7

[6] Hunsballe JM, Djurhuus JC. Clinical options for imipramine in the management of urinary incontinence. Urol Res. 2001;29: 118–125. 11396729 10.1007/s002400100175

[7] Miller KL. Stress urinary incontinence in women: review and update on neurological control. J Wom Health (Larchmt). 2005;14: 595–608. 16181016 10.1089/jwh.2005.14.595

[8] Dugan SE, Fuller MA. Duloxetine: a dual reuptake inhibitor. Ann Pharmacother. 2004;38: 2078–2085. 15522980 10.1345/aph.1E084

[9] Müller N, Schennach R, Riedel M, Möller HJ. Duloxetine in the treatment of major psychiatric and neuropathic disorders. Exp Rev Neurother. 2008;8: 527–536. 10.1586/14737175.8.4.527 18416656

[10] Jost WH, Marsalek P. Duloxetine in the treatment of stress urinary incontinence. Ther Clin Risk Manag. 2005;1: 259–264. 18360568 PMC1661641

[11] NICE (National Institute for Health and Clinical Excellence). Clinical guideline 171: Urinary incontinence. London, UK; 2013.

[12] Boudes M, Uvin P, Kerselaers S, Vennekens R, Voets T, De Ridder D. Functional characterization of a chronic cyclophosphamide-induced overactive bladder model in mice. Neurourol Urodynam. 2011;30: 1659–1665. 10.1002/nau.21180 21717507

[13] Mucignat-Caretta C, Bondi’ M, Caretta A. Endocrine status affects bladder size and post-void residual urinary volume in mice. Horm Behav. 2004A;46: 11–18. 15215037 10.1016/j.yhbeh.2004.02.004

[14] Cavaggioni A, Mucignat-Caretta C, Redaelli M. Mice Recognize Recent Urine Scent Marks by the Molecular Composition. Chem Sens. 2008;33: 655–663. 10.1093/chemse/bjn035 18603651

[15] Mogi K, Shimokawa Y, Nagasawa M, Kikusui T. Effects of sex and rearing environment on imipramine response in mice. Psychopharmacology (Berl). 2012;224: 201–208. 10.1007/s00213-012-2821-y 22868412

[16] Cottingham C, Li X, Wang Q. Noradrenergic antidepressant responses to desipramine in vivo are reciprocally regulated by arrestin3 and spinophilin. Neuropharmacology. 2012;62: 2354–2362. 10.1016/j.neuropharm.2012.02.011 22369787 PMC3314098

[17] Marlatt MW, Lucassen PJ, van Praag H. Comparison of neurogenic effects of fluoxetine, duloxetine and running in mice. Brain Res. 2010;1341, 93–99. 10.1016/j.brainres.2010.03.086 20381469 PMC2884062

[18] Lantz RJ, Gillespie TA, Rash TJ, Kuo F, Skinner, Kuan HY, et al Metabolism, excretion, and pharmacokinetics of duloxetine in healthy human subjects. Drug Metab Disposit. 2003;31: 1142–1150. 12920170 10.1124/dmd.31.9.1142

[19] Kuo F, Gillespie TA, Kulanthaivel P, Lantz RJ, Ma TW, Nelson DL, et al Synthesis and biological activity of some known and putative duloxetine metabolites. Bioorg Medic Chem Lett. 2004;14, 3481–3486. 15177457 10.1016/j.bmcl.2004.04.066

[20] Ricatti MJ, Alfie LD, Lavoie EG, Sévigny J, Schwarzbaum PJ., Faillace MP. Immunocytochemical localization of NTPDases1 and 2 in the neural retina of mouse and zebrafish. Synapse. 2009;63: 291–307. 10.1002/syn.20605 19116950

[21] Beckel JM, Holstege G. Neurophysiology of the lower urinary tract. Handb Exp Pharmacol. 2011;202: 149–169. 10.1007/978-3-642-16499-6_8 21290226

[22] Andersson KE, Pehrson R. CNS involvement in overactive bladder: pathophysiology and opportunities for pharmacological intervention. Drugs. 2003;63: 2595–2611. 14636079 10.2165/00003495-200363230-00003

[23] Seth JH, Panicker JN, Fowler CJ. The neurological organization of micturition. Handb Clin Neurol. 2013;117: 111–117. 10.1016/B978-0-444-53491-0.00010-9 24095120

[24] Coyne KS, Wein A, Nicholson S, Kvasz M, Chen CI, Milsom I. Comorbidities and personal burden of urgency urinary incontinence: a systematic review. Int J Clin Pract. 2013;67: 1015–1033. 10.1111/ijcp.12164 24073974

[25] Smith AL, Wein AJ. Urinary incontinence: pharmacotherapy options. Ann Med. 2011;43: 461–476. 10.3109/07853890.2011.564203 21639723

[26] Szigeti GP, Somogyi GT, Csernoch L, Széll EA. Age-dependence of the spontaneous activity of the rat urinary bladder. J Muscle Res Cell Motil. 2005;26: 23–29. 16025204 10.1007/s10974-005-9003-z

[27] de Groat WC. Anatomy of the central neural pathways controlling the lower urinary tract. Eur Urol 1998;34: 2–5. 9705544 10.1159/000052265

[28] de Groat WC. Integrative control of the lower urinary tract: preclinical perspective. Br J Pharmacol. 2006;147 Suppl 2: S25–S40. 16465182 10.1038/sj.bjp.0706604 PMC1751498

[29] Fowler CJ, Griffiths D, de Groat WC. The neural control of micturition. Nat Rev Neurosci. 2008;9: 453–466. 10.1038/nrn2401 18490916 PMC2897743

[30] Tai C, Wang J, Jin T, Wang P, Kim SG, Roppolo JR, et al Brain switch for reflex micturition control detected by FMRI in rats. J Neurophysiol. 2009;102: 2719–2730. 10.1152/jn.00700.2009 19741099 PMC2777821

[31] Drake MJ, Fowler CJ, Griffiths D, Mayer E, Paton JF, Birder L. Neural control of the lower urinary and gastrointestinal tracts: supraspinal CNS mechanisms. Neurourol. Urodynam. 2010;29: 119–127. 10.1002/nau.20841 20025025

[32] Griffiths D, Tadic SD. Bladder control, urgency, and urge incontinence: evidence from functional brain imaging. Neurourol. Urodynam. 2008;27: 466–474. 10.1002/nau.20549 18092336

[33] Griffiths DJ, Fowler CJ. The micturition switch and its forebrain influences. Acta Physiol. 2013;207: 93–109. 10.1111/apha.12019 23164237

[34] Schuessler B. What do we know about duloxetine's mode of action? Evidence from animals to humans. BJOG. 2006;113 Suppl 1: 5–9. 16529563 10.1111/j.1471-0528.2006.00877.x

[35] Burgard EC, Fraser MO, Thor KB. Serotonergic modulation of bladder afferent pathways. Urology. 2003;62: 10–15. 14550832 10.1016/s0090-4295(03)00590-9

[36] Griffiths D, Derbyshire S, Stenger A, Resnick N. Brain control of normal and overactive bladder. J Urol. 2005;174: 1862–1867. 16217325 10.1097/01.ju.0000177450.34451.97

[37] Maggi CA, Borsini F, Lecci A, Giuliani S, Meli P, Gragnani L et al Effect of acute or chronic administration of imipramine on spinal and supraspinal micturition reflexes in rats. J Pharmacol Exp Ther. 1989;248: 278–285. 2521514

[38] Di Rezze S, Frasca V, Inghilleri M, Durastanti V, Cortese A, Giacomelli E et al Duloxetine for the treatment of overactive bladder syndrome in multiple sclerosis: a pilot study. Clin Neuropharmacol. 2012;35: 231–234. 10.1097/WNF.0b013e3182613dce 22751087

[39] Jost WH. Urological problems in Parkinson's disease: clinical aspects. J Neur Transm. 2013;120: 587–591. 10.1007/s00702-012-0914-8 23196979

[40] Lamin E, Smith AL. Urologic agents for treatment of bladder dysfunction in neurologic disease. Cur Treat Opt Neurol. 2014;16: 280. 10.1007/s11940-013-0280-3 24464489

[41] Kakizaki H, Kita M, Wada N. Models for sensory neurons of dorsal root ganglia and stress urinary incontinence. Neurourol Urodyn. 2011;30: 653–657. 10.1002/nau.21138 21661009

[42] Mucignat-Caretta C, Bondi’ M, Caretta A. Animal models of depression: olfactory lesions affect amygdala, subventricular zone and aggression. Neurobiol Dis. 2004B;16: 386–395. 15193295 10.1016/j.nbd.2004.03.007

[43] Golubeva AV, Zhdanov AV, Mallel G, Dinan TG, Cryan JF. The mouse cyclophosphamide model of bladder pain syndrome: tissue characterization, immune profiling, and relationship to metabotropic glutamate receptors. Physiol Rep. 2014;2: e00260 10.1002/phy2.260 24760514 PMC4002240

