# Supplementary material for: Serotonin and Noradrenaline Reuptake Inhibitors Improve Micturition Control in Mice
Source: PLoS One. 2015 Mar 26;10(3):e0121883. doi: 10.1371/journal.pone.0121883 (PMC4374881; doi:10.1371/journal.pone.0121883)
Supplement: S1 Data — (PDF) [file pone.0121883.s001.pdf]

RAW DATA FOR FIG. 1

A) first voiding latency (sec)

CTRL	CYP40
1800	501
1745	376
1664	513
1161	746
1414	444
	563
	456

B) spots Volume (ul)

CTRL	CYP40
66	138
111	106
117	373
113	214
95	322
	115
	216

C) number of spots (n°)

CTRL	CYP40
2	9
4	11
2	13
3	7
6	6
	11
	14

D) body weight (%)

CYP40	Day 0	Day 2	Day 4	Day 6
	100	113,04348	113,04348	113,04348
	100	102,94118	100	104,90196
	100	100	98,888889	100
	100	100,9434	98,113208	100
	100	100	96,153846	96,923077
	100	100	96,153846	100
	100	100	97	100
CTRL	Day 0	Day 2	Day 4	Day 6
	100	101,06383	101,06383	108,51064
	100	97,959184	97,959184	98,979592
	100	92,156863	93,137255	93,137255
	100	99,019608	96,078431	101,96078
	100	102,22222	100	103,33333

Actual Weight (grams)

CYP40	Day 0	Day 2	Day 4	Day 6
	46	52	52	52
	51	52,5	51	53,5
	45	45	44,5	45
	53	53,5	52	53
	65	65	62,5	63
	52	52	50	52
	50	50	48,5	50
CTRL	Day 0	Day 2	Day 4	Day 6
	47	47,5	47,5	51
	49	48	48	48,5
	51	47	47,5	47,5
	51	50,5	49	52
	45	46	45	46,5
