# Supplementary material for: Serotonin and Noradrenaline Reuptake Inhibitors Improve Micturition Control in Mice
Source: PLoS One. 2015 Mar 26;10(3):e0121883. doi: 10.1371/journal.pone.0121883 (PMC4374881; doi:10.1371/journal.pone.0121883)
Supplement: S2 Data — (PDF) [file pone.0121883.s002.pdf]

RAW DATA FOR FIGURE 2

| Saline | A) first voiding latency (sec) 0 h | 24 h | 48 h | B) spots Volume (ul) 0 h | 24 h | 48 h | C) number of spots (n°) 0 h | 24 h | 48 h |
|---|---|---|---|---|---|---|---|---|---|
| **S1** | 630 | 178 | 247 | 207 | 515 | 136 | 19 | 16 | 17 |
| **S2** | 532 | 299 | 476 | 180 | 189 | 379 | 9 | 7 | 9 |
| **S3** | 492 | 229 | 621 | 204 | 202 | 728 | 10 | 7 | 7 |
| **S4** | 72 | 188 | 119 | 406 | 207 | 371 | 9 | 6 | 9 |
| **S5** | 70 | 38 | 98 | 159 | 220 | 355 | 8 | 7 | 6 |
| **S6** | 340 | 652 | 370 | 215 | 200 | 321 | 9 | 6 | 9 |
| **S7** | 267 | 382 | 421 | 288 | 670 | 378 | 18 | 19 | 14 |
| **Imipramine** | | | | | | | | | |
| **I1** | 200 | 124 | 307 | 217 | 582 | 86 | 8 | 6 | 2 |
| **I2** | 80 | 1200 | 1200 | 76 | 386 | 0 | 6 | 3 | 0 |
| **I3** | 50 | 744 | 1200 | 1077 | 17 | 0 | 10 | 7 | 0 |
| **I4** | 185 | 637 | 618 | 577 | 323 | 8 | 21 | 13 | 1 |
| **I5** | 723 | 1200 | 1200 | 78 | 59 | 0 | 8 | 4 | 0 |
| **I6** | 814 | 1106 | 1080 | 74 | 194 | 11 | 4 | 3 | 1 |
| **I7** | 112 | 722 | 997 | 27 | 59 | 64 | 6 | 4 | 1 |
| **Desipramine** | | | | | | | | | |
| **D1** | 720 | 760 | 1200 | 332 | 18 | 22 | 6 | 2 | 3 |
| **D2** | 820 | 500 | 559 | 151 | 29 | 57 | 37 | 4 | 5 |
| **D3** | 312 | 496 | 1200 | 102 | 20 | 61 | 8 | 3 | 6 |
| **D4** | 525 | 680 | 693 | 199 | 36 | 254 | 18 | 3 | 10 |
| **D5** | 1000 | 545 | 1200 | 116 | 27 | 296 | 8 | 1 | 2 |
| **D6** | 55 | 37 | 1200 | 222 | 26 | 0 | 4 | 2 | 0 |
| **D7** | 229 | 372 | 854 | 350 | 27 | 0 | 9 | 3 | 0 |
| **Duloxetine** | | | | | | | | | |
| **Du1** | 229 | 1200 | 895 | 275 | 34 | 0 | 12 | 2 | 0 |
| **Du2** | 295 | 197 | 450 | 370 | 28 | 25 | 6 | 2 | 1 |
| **Du3** | 572 | 1200 | 360 | 236 | 28 | 53 | 19 | 1 | 1 |
| **Du4** | 89 | 18 | 470 | 87 | 23 | 58 | 8 | 1 | 1 |
| **Du5** | 836 | 1200 | 813 | 198 | 18 | 0 | 5 | 1 | 0 |
| **Du6** | 525 | 1200 | 1200 | 45 | 20 | 16 | 7 | 1 | 1 |
| **Du7** | 421 | 850 | 1200 | 209 | 41 | 57 | 32 | 2 | 2 |
| **4-Hydroxy-Duloxetine** | | | | | | | | | |
| **4H1** | 35 | 1200 | 1200 | 290 | 10 | 10 | 8 | 2 | 1 |
| **4H2** | 215 | 1200 | 1055 | 649 | 283 | 37 | 19 | 10 | 2 |

| | | | | | | | | | |
|---|---|---|---|---|---|---|---|---|---|
| **4H3** | 452 | 461 | 626 | 752 | 642 | 0 | 28 | 14 | 0 |
| **4H4** | 433 | 669 | 1200 | 654 | 11 | 10 | 14 | 3 | 2 |
| **4H5** | 862 | 1033 | 996 | 275 | 9 | 0 | 7 | 1 | 0 |
| **4H6** | 229 | 897 | 1200 | 134 | 13 | 8 | 6 | 1 | 1 |
| **4H7** | 551 | 699 | 921 | 258 | 16 | 11 | 11 | 2 | 2 |
