# Supplementary material for: Serotonin and Noradrenaline Reuptake Inhibitors Improve Micturition Control in Mice
Source: PLoS One. 2015 Mar 26;10(3):e0121883. doi: 10.1371/journal.pone.0121883 (PMC4374881; doi:10.1371/journal.pone.0121883)
Supplement: S3 Data — (PDF) [file pone.0121883.s003.pdf]

RAW DATA FOR FIGURE 3 - individual measurement in µm of the thickness of the urothelium

group	h1	h2	h3	h4	h5	h6	h7	h8	h9	h10	h11	h12	h13	h14	h15	mean/slice µm	subject
droxy-dulox	204	84	33	163	35	22	26	42	43	63	103	33	18	35	30	62,27	5
droxy-dulox	78	96	87	185	236	42	16	34	18	29	27	69	118	62	18	74,33	4
droxy-dulox	25	32	47	45	46	36	22	21	44	27	37	102	63	34	28	40,60	3
droxy-dulox	42	90	38	40	73	41	28	53	21	34	21	37	29	33	42	41,47	3
droxy-dulox	62	50	36	33	54	21	54	28	39	49	66	40	118	30	18	46,53	6
droxy-dulox	30	24	21	37	32	39	56	32	43	37	29	65	23	83	62	40,87	1
droxy-dulox	34	67	31	13	33	15	65	50	71	23	14	117	55	31	73	46,13	6
droxy-dulox	55	21	50	23	34	85	41	26	46	12	46	43	38	54	44	41,20	1
droxy-dulox	80	53	62	74	33	35	28	43	57	87	38	77	32	13	72	52,27	7
droxy-dulox	40	77	27	63	35	106	113	78	144	173	232	152	66	64	62	95,47	2
droxy-dulox	*24*	*25*	*30*	*48*	*265*	*310*	*233*	*221*	*79*	*30*	*38*	*40*	*51*	*232*	*25*	*110,07*	2
imipramine	38	32	53	39	57	75	61	60	51	42	48	29	67	54	65	51,40	2
imipramine	133	52	47	87	51	27	173	35	49	67	124	91	130	111	47	81,60	2
imipramine	67	50	18	41	115	79	36	99	54	73	23	59	94	78	48	62,27	2
imipramine	119	55	46	96	51	116	175	36	37	52	128	133	76	90	44	83,60	3
imipramine	170	37	155	52	46	44	59	44	16	27	50	45	81	29	68	61,53	1
imipramine	44	30	48	65	65	18	77	50	33	65	88	155	147	72	206	77,53	7
imipramine	23	22	22	41	36	22	60	20	53	71	37	41	31	62	180	48,07	5
imipramine	34	49	77	54	222	29	28	85	68	23	56	75	38	52	46	62,40	1
imipramine	59	52	44	49	51	74	42	93	84	70	62	55	88	37	98	63,87	6
imipramine	57	59	35	58	33	48	52	61	36	158	68	40	24	48	50	55,13	4
imipramine	36	57	57	75	51	35	30	298	49	24	54	42	85	155	58	73,73	4
desipramine	111	40	27	117	35	62	22	79	22	104	129	55	60	50	62	65,00	7
desipramine	116	64	66	113	199	67	34	26	38	142	43	58	94	98	78	82,40	6
desipramine	148	135	134	70	76	72	112	62	39	34	38	55	61	36	44	74,40	1
desipramine	94	146	75	43	39	35	64	42	78	46	46	36	29	28	33	55,60	7
desipramine	70	81	56	66	88	52	55	44	35	31	29	60	64	80	36	56,47	1
desipramine	49	34	62	153	44	39	70	135	39	128	84	100	148	44	35	77,60	2
desipramine	33	31	147	149	136	63	35	134	35	148	89	27	35	62	38	77,47	5
desipramine	66	145	76	54	66	184	92	47	81	50	56	53	55	56	177	83,87	4
desipramine	60	73	100	62	36	74	63	61	52	48	36	81	54	34	47	58,73	6
desipramine	106	40	25	36	34	42	72	118	91	51	38	30	31	77	83	58,27	3
salina	47	42	46	79	92	97	107	46	42	35	84	41	72	74	39	62,87	5
salina	42	59	40	30	26	53	52	52	55	70	127	93	75	61	41	58,40	2
salina	56	58	39	114	64	37	41	56	35	67	60	70	63	59	39	57,20	7
salina	*116*	*142*	*162*	*156*	*193*	*149*	*118*	*92*	*120*	*76*	*109*	*124*	*133*	*162*	*126*	*131,87*	4
salina	69	67	82	88	114	133	109	59	40	17	46	49	82	148	94	79,80	7
salina	80	69	75	93	111	71	94	103	105	74	77	55	117	83	110	87,80	6
salina	73	72	61	81	65	58	36	142	70	124	81	110	96	98	109	85,07	2
salina	79	66	102	62	70	65	146	94	145	78	90	108	47	96	71	87,93	3
salina	88	101	68	124	74	87	76	123	96	76	62	65	61	82	77	84,00	7
salina	119	117	104	75	76	70	71	56	39	45	40	42	63	91	63	71,40	1

duloxetine	36	63	54	66	58	38	47	78	54	64	43	72	49	43	41	53,73	3
duloxetine	73	70	74	45	104	86	102	96	77	75	72	81	84	96	173	87,20	7
duloxetine	83	71	96	65	68	63	57	80	95	75	73	97	102	87	73	79,00	7
duloxetine	137	76	64	90	94	103	112	111	115	110	129	138	109	123	112	108,20	4
duloxetine	61	53	68	58	61	65	60	45	61	57	52	56	95	79	56	61,80	1
duloxetine	77	68	67	71	56	45	73	60	48	40	38	71	49	67	61	59,40	6
duloxetine	53	41	45	53	41	48	61	59	55	56	51	54	71	91	64	56,20	2
duloxetine	91	93	69	72	64	37	50	45	44	52	74	78	95	106	111	72,07	5
control	45	34	12	52	43	32	60	45	62	45	28	16	44	59	35	40,80	5
control	39	37	14	20	28	36	31	42	26	29	24	32	47	62	62	35,27	7
control	29	67	36	35	56	37	35	25	60	53	54	58	36	30	58	44,60	7
control	33	41	58	51	56	65	60	39	32	34	54	49	28	48	63	47,40	3
control	28	27	34	49	72	36	42	26	44	37	28	45	66	21	48	40,20	1
control	19	21	38	32	30	41	26	41	28	32	42	44	66	51	64	38,33	6
control	33	18	28	53	62	60	54	29	30	45	42	35	42	30	47	40,53	2
control	27	35	40	29	34	29	38	86	50	29	41	34	14	48	42	38,40	6
control	25	41	75	27	25	15	32	30	22	16	15	21	23	32	28	28,47	5
control	47	35	45	18	33	35	18	29	27	43	29	33	40	32	33	33,13	5
control	18	38	15	21	16	36	27	34	65	26	46	40	63	53	27	35,00	4
control	41	36	41	76	32	32	35	50	52	31	48	50	36	25	56	42,73	3

an/slice µm		mean/slice µm		mean/slice µm		mean/slice µm		mean/slice µm		mean/slice µm	
control	subject	saline	subject	mipramine	subject	esipramine	subject	duloxetine	subject	-duloxetine	subject
40,80	5	62,87	5	51,40	2	65,00	7	53,73	3	62,27	5
35,27	7	58,40	2	81,60	2	82,40	6	87,20	7	74,33	4
44,60	7	57,20	7	62,27	2	74,40	1	79,00	7	40,60	3
47,40	3	131,87	4	83,60	3	55,60	7	108,20	4	41,47	3
40,20	1	79,80	7	61,53	1	56,47	1	61,80	1	46,53	6
38,33	6	87,80	6	77,53	7	77,60	2	59,40	6	40,87	1
40,53	2	85,07	2	48,07	5	77,47	5	56,20	2	46,13	6
38,40	6	87,93	3	62,40	1	83,87	4	72,07	5	41,20	1
28,47	5	84,00	7	63,87	6	58,73	6			52,27	7
33,13	5	71,40	1	55,13	4	58,27	3			95,47	2
35,00	4			73,73	4					110,07	2
42,73	3										

mean	subject	mean	subject	mean	subject	mean	subject	mean	subject	mean	subject
45,07	(mean) 3	71,73	(mean) 2	61,97	(mean) 1	65,43	(mean) 1	83,10	(mean) 7	41,03	(mean) 1
34,13	(mean) 5	73,67	(mean) 7	65,09	(mean) 2	70,57	(mean) 6	61,80	1	102,77	(mean) 2
38,37	(mean) 6	71,40	1	64,43	(mean) 4	60,30	(mean) 7	56,20	2	41,03	(mean) 3
39,93	(mean) 7	87,93	3	83,60	3	77,60	2	53,73	3	46,33	(mean) 6
40,20	1	131,87	4	48,07	5	58,27	3	108,20	4	74,33	4
40,53	2	62,87	5	63,87	6	83,87	4	72,07	5	62,27	5
35,00	4	87,80	6	77,53	7	77,47	5	59,40	6	52,27	7

control	saline	imipramine	desipramine	duloxetine	4-OH-duloxetine
