# Supplementary material for: Serotonin and Noradrenaline Reuptake Inhibitors Improve Micturition Control in Mice
Source: PLoS One. 2015 Mar 26;10(3):e0121883. doi: 10.1371/journal.pone.0121883 (PMC4374881; doi:10.1371/journal.pone.0121883)
Supplement: S4 Data — (PDF) [file pone.0121883.s004.pdf]

RAW DATA FOR SUPPORTING FILE S1

Calibration curve

ul of urine	squared pixels
2	1155
5	4120
10	7913
25	12303
50	23788
100	42512
250	121573
350	173305
500	214496
750	335416

Equation

$y = 445.39x + 2710.3$

R2 = 0.9964
