# Supplementary figures and images for: Serotonin and Noradrenaline Reuptake Inhibitors Improve Micturition Control in Mice
Source: PLoS One. 2015 Mar 26;10(3):e0121883. doi: 10.1371/journal.pone.0121883 (PMC4374881; doi:10.1371/journal.pone.0121883)

Calibration curve

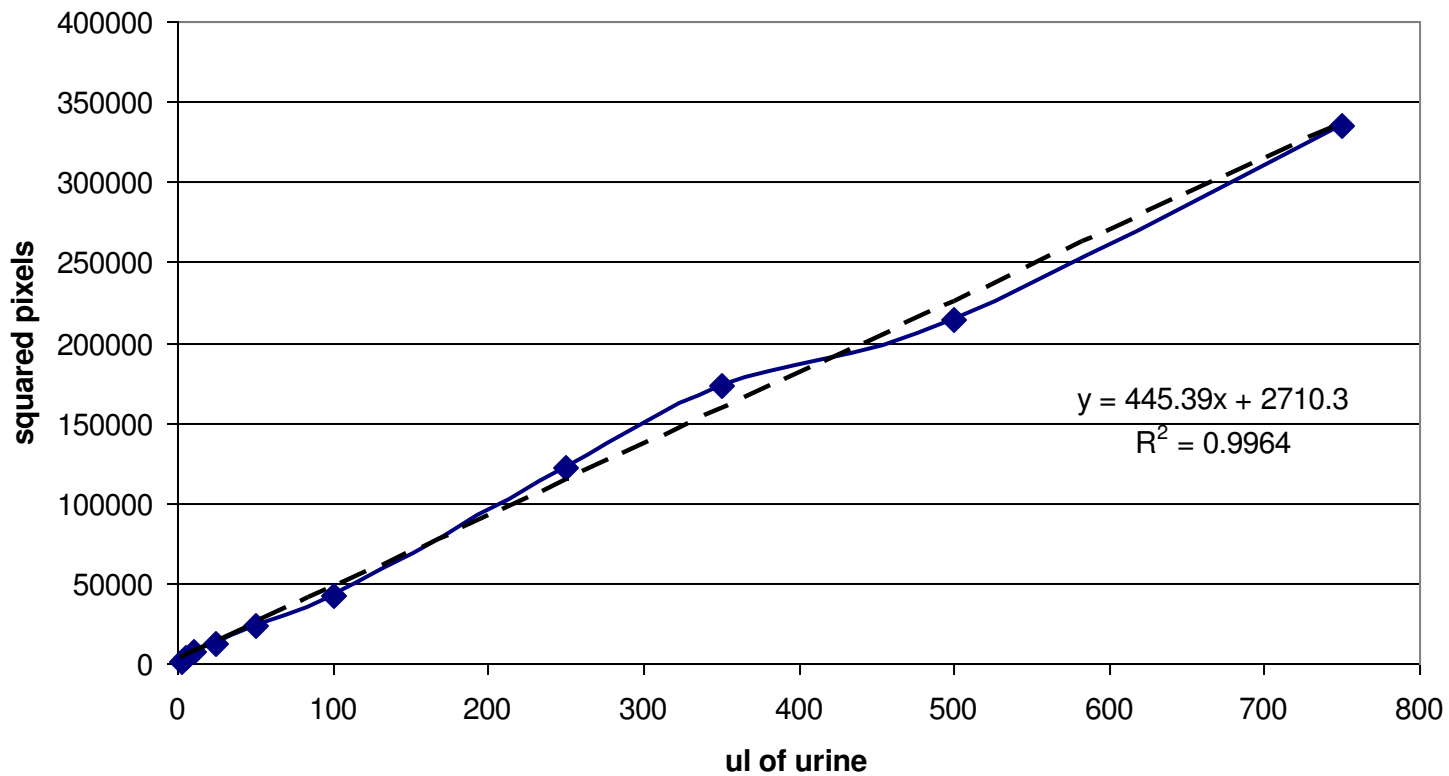

Supplement: S1 Fig — Different volumes of male mouse urine (from 1 to 750 microliters) were spotted on the Benchguard paper sheet (the same type used for urine drop analysis) and processed in the same way: they were left for 48 hours at room temperature, then UV transilluminated and photographed. The total number of pixels within urine spots was calculated with ImageJ software, then converted into volume of voided urine according to the equation: y = 445.39x + 2710.3. This gives a linear fit R2 = 0.9964, according to the calibration curve. (PDF) [file pone.0121883.s005.pdf]

# Experimental Design

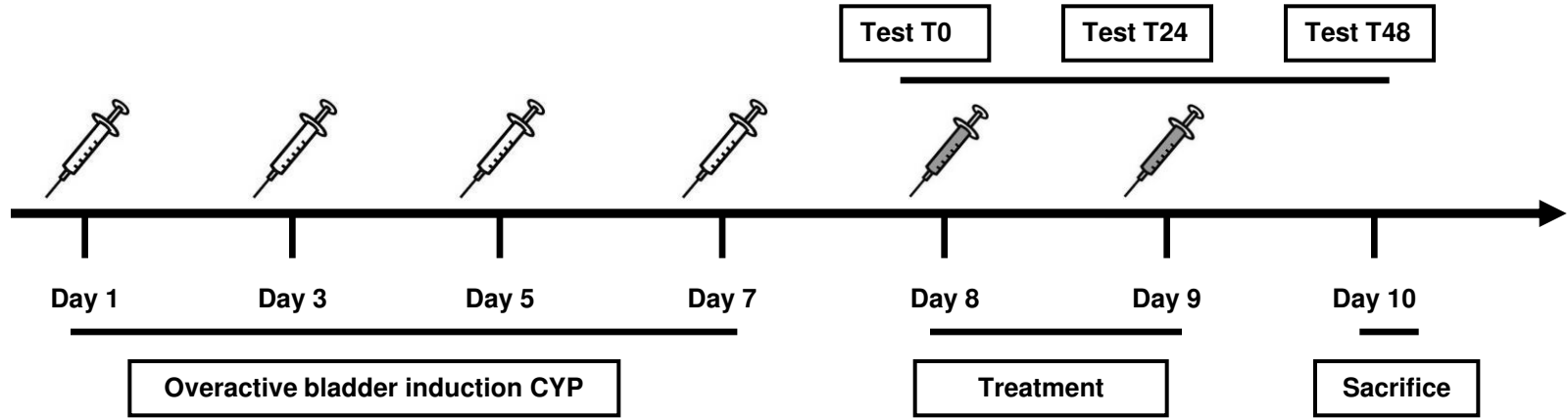

Supplement: S2 Fig — Mice were injected with CYP (40 mg/kg), one injection every 48 hours, for four times. 24 hours after the last CYP injection mice were tested for micturition (T0) and then were given a control (saline) or antidepressant injection (see text for dosages). 24 hours later, they were tested again (T24) and then were given the second injection. After additional 24 hours, they were tested for micturition behavior for the third time (T48). (PDF) [file pone.0121883.s006.pdf]
